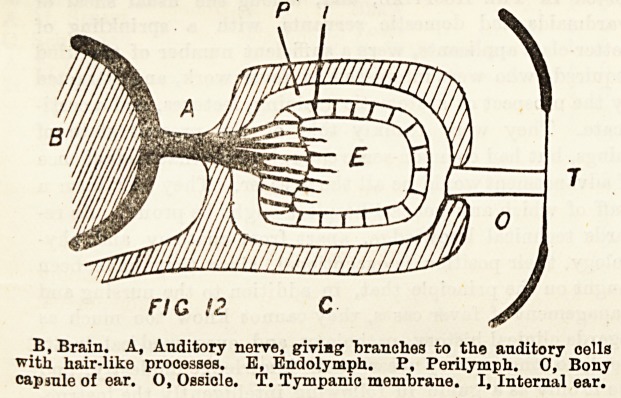# The Hospital Nursing Supplement

**Published:** 1895-11-23

**Authors:** 


"The Hospital" Nursing Mirror.

Being the Extra Nursing Supplement of "The Hospital" Newspaper.

[Contributions for this Supplement should be addressed to the Editor, The Hospital, 428, Strand, London, W.C., and should have the word "Nursing" plainly written in left-hand top corner of the envelope.]

### News from the Nursing World.

### CHEYNE HOSPITAL.

The Princess of Wales is president of the Hospital for Sick and Incurable Children, Cheyne Walk, Chelsea, of which the announcement of the annual exhibition of needlework appears in our columns this week. On December 3rd and 4th guests are invited to see the work which the patients have accomplished during the year, and doubtless many visitors will take the opportunity of visiting this excellent institution. There is great variety in the beautiful work done by the incurable children, and great patience must have been devoted by their voluntary instructors to making them so expert. It is expressly stated that the needlework is not for sale, in the invitations issued by the lady superintendent, Miss Elam.

### NURSES FOR ASHANTI.

In connection with the departure of troops, medical stores, &c., for Ashanti, it is announced that orders have been received for Mrs. Gray, acting superintendent of nurses, to hold herself in readiness for departure early in December. She will be accompanied by several nursing Sisters. Mrs. Gray is a Dame of the Order of the Royal Red Cross, and had experience in the war in Zululand and in Egypt.

### SAD DEATH OF A PRIVATE PATIENT.

The circumstances attending the suicide of a young man at Ealing last week appeared from the press report to demand explanation, for it was stated that the patient, delirious with typhoid fever, was sitting on the edge of the bed, Margaret Veitch, a trained nurse from St. Faith's Nursing Institution, Ealing, being with him. It seems, however, from later accounts, that the young man was able to be dressed, and therefore his sitting up was not so surprising as it sounded when he was described as "a typhoid patient." He appeared to become suddenly violent, walked to the door, locked it, and attempted to blow his brains out with a revolver. As this missed fire, the nurse secured the key, opened the door, and ran into the passage shouting for help, and the unfortunate patient meanwhile succeeded in shooting himself dead. His mother, sister, and another nurse responded to the nurse's cry, but entered the room too late to prevent the catastrophe. The unusual height and strength of the young man rendered it impossible for a woman to manage him single-handed, and if immediate fears had been entertained of his becoming uncontrollable, it is unlikely that he would have been left, even temporarily, with only one attendant.

### BIRMINGHAM WOMEN'S HOSPITAL.

The Countess of Dudley spoke with great cordiality of the excellent work done at the Birmingham and Midland Hospital the other day on the occasion of her opening a sale of work in aid of the funds of the institution. Increased subscriptions are needed for maintenance and to clear off the outstanding debt. A convalescent home forms a valuable adjunct of this charity, and is greatly appreciated by the patients.

### AN APPEAL FROM NEWCASTLE.

An urgent appeal for the Newcastle Cathedral Nurse and Loan Society has been made by the Vicar, who draws attention to a decrease in financial support at a season when such aid is particularly needed. The invalid kitchen, which has proved such a valuable auxiliary to the nurse's work, requires fresh subscriptions at once, and in a city like Newcastle these will doubtless be forthcoming as soon as the urgency of the case is made known.

### MALE NURSES.

It is with regret that we learn that the scheme for training male nurses has been abandoned at the Seamen's Hospital, Greenwich. A perusal of our columns will show that a large number of men are desirous of receiving adequate training, a fact which was questioned at the meeting at which the proposal was rejected. The total absence of training amongst male nurses in England is principally responsible for the unpopularity from which they suffer, and, therefore, their employment is frequently most unsatisfactory. In spite of this, there is a steady and continuous demand for a certain number of male nurses, and that no training is to be procured for them shows both a lack of public spirit and a selfishness which is discreditable to our institutions, which, after all, depend on the public for support, and are willing recipients of its bounty. We go so far as to believe that, in the case of a hospital possessing certain facilities, as at the Seamen's Hospital, but which would necessarily incur extra expense, special funds could be secured to render the adoption of the scheme advantageous to the institution, as well as most desirable in the interests of the public. If the introduction of a limited number of male probationers into hospitals is possible in other countries, we fail to see why it is an insuperable difficulty in England.

### HOLIDAYS DISCOURAGED.

The chairman of the Bream Board of Guardians is reported to have told the newly-appointed infirmary nurse that it was not convenient for her to have a holiday before entering upon her duties, promising somewhat vaguely that she should have one later on. She pointed out that during the past twelve months she had been at the Isolation Hospital and had no holiday, petitioning for a short one before commencing her new work. This reasonable request was refused to the nurse, who was unanimously elected to fill an important and responsible position without her much-needed holiday. The ratepayers would do well to see that their representatives on the Board have some elementary knowledge of the consideration due to officials, whose usefulness and health must suffer when holidays are too long postponed.

### THE INTRUSION OF HALF-TRAINED NURSES.

We need not hesitate to express our regret at finding it stated in the Annual Report of the Lincolnshire Nursing Association that such a body, which employs the half-trained sort of people who go by the name of "cottage nurses," has become affiliated to the Queen Victoria Jubilee Institute, and that Miss Peters, reporting in favour of the system of employing two classes of nurses, considers that "Lincolnshire is extremely satisfactory." The same report announces that County Council funds are being obtained for the purpose of "training" these women. There can be no doubt that such patronage and encouragement of a faulty and pernicious system is likely to do great harm to properly trained nurses, besides the untold and undiscoverable injury inflicted by the system upon the sick poor. For many years back the leaders of the nursing profession have struggled to ensure that those who undertake the work shall have a proper knowledge of their duties, a knowledge which can only be obtained by a prolonged training under proper teachers, and it is saddening in the extreme to find that so little is the interest shown by the public in matters which have been undertaken purely for their benefit, that they acquiesce in, and even accept as a good deed, this cheap production of half-trained nurses, which must, if it is persisted in, have the effect of bringing down the standard of nursing throughout the whole country both for rich and poor. We, and all who have had the true interests both of the public and the nurses near at heart, have always aimed at making no class distinctions, but at providing all alike with nurses who were fully trained. Class distinctions have always been hateful to us, and we have maintained that we ought not to wait for death, the great leveller, to wipe them out, but that in the presence of sickness all should be equal. If, however, these associations for cheap nursing are to have their way all this must be altered, and in sickness as in health the poor must put up with the second rate. It certainly never can be borne that these half-trained women can be accepted as the equals of those who have qualified themselves for responsible work by undergoing a proper training. The almost necessary outcome, then, will be that two distinct classes of nurses will come into existence; but none will regret more than we shall that the sacrifices of so many noble women should be thrown away, and that, in consequence of a demand for cheap charity, their efforts to make sickness a passport to equality should be brought to naught, and that again the old system which it was hoped was dying should be revived, of regarding anything as "good enough for the poor."

### NURSING AT LISBURN.

Attention was called by a coroner's jury, the other day, to the inadequate night nursing at Lisburn Workhouse. Including lunatics, there are sixty-five patients distributed in seven wards, and they are left during the night in the sole charge of a woman, said to be considerably over sixty years of age. Whilst she was in attendance at one death-bed a second patient died unexpectedly, and at the subsequent inquest a rider to the verdict stated that "the jury desire to draw the attention of the Guardians to the inadequate night nursing in the infirmary of the workhouse."

### QUEEN'S NURSES AT INVERNESS.

The introduction of a Queen's nurse at Inverness four years ago has resulted in much useful work being done among the sick poor in their own homes. It is desirable that a second nurse should be engaged as soon as the funds permit, for the work is steadily increasing. Perhaps the Parish Council might be prevailed upon to assist, for it has been recently announced that this body requires a trained nurse for outdoor work amongst the sick. As their proposal to engage one who should combine district nursing with attendance on the poorhouse in-patients has been negatived by the Local Government Board, the Parish Council might fitly confer with the Scotch branch of the Queen's Institute on the matter of outdoor nursing.

### A NURSE OUT OF WORK.

The New York press, in reporting the attempted suicide of an English-trained nurse in that city, asserts that the unhappy woman had been unsuccessful in finding employment. She had, apparently, left a delicate husband and four children in the old country, under the impression that she could make a living for them and herself in the States. The intervention of two men defeated her attempt to drown herself, and probably other people have befriended her ere this. The occurrence serves to emphasise what has been frequently stated in The Hospital respecting the folly of nurses leaving England without first getting reliable information as to the prospects which await them elsewhere. The United States, as well as many of our colonies, now possess numerous schools and train a sufficient supply of nurses for themselves.

### SHORT ITEMS.

The Guardians have decided to appoint an additional trained nurse at Bridgend Workhouse.—At Cockermouth a trained district nurse has been selected by a committee of ladies; no fees will be accepted for her services, but patients able to subscribe to the society will be expected to do so.—A salary of £15 per annum is offered by the Havant Guardians for a "sick ward attendant and nurse" for their workhouse!—Greengates and Apperley Nursing Fund is £18 the richer for an entertainment held for the benefit of its funds at the Board school.—The Trim Board of Guardians have decided to appoint a trained night nurse with a knowledge of midwifery to their workhouse.—The *Britannia* brought home 97 sick from India, all being taken to the Royal Victoria Hospital at Netley.—The last issue of the *Nurses' Journal* contains Dr. Louis Parkes' address, given at Bedford College; also reports of meetings, and lists of members of the Royal British Nurses' Association, of which it is the official organ.—The Poor-house Committee at Dumbarton have a third time refused to appoint trained nurses in their hospital.—The Romford District Council have decided to add another nurse to their staff at a salary of £30 per annum.—At the annual meeting of the Selkirk branch of the Queen's Jubilee Institute it was stated that Nurse Rumsey had done her dutes to the entire satisfaction of the committee.—The institution of a trained district nurse at Wombwell is contemplated, it being resolved at a recent meeting to endeavour to secure for this movement the friendly interest and support of the Miners' societies.

### Elementary Physiology for Nurses.

By C. F. Marshall, late Surgical Registrar Hospital for Sick Children, Great Ormond Street.

XVI.—THE SENSE OF HEARING (*concluded*).

*The Mechanism of Hearing.*—If a tuning-fork is struck and placed on the head sound is heard very distinctly. The vibrations are communicated directly to the bones of the head and so to the perilymph, the internal ear of the endolymph. There the vibrations affect the auditory nerve endings and give rise to the sense of sound through the auditory nerve to the brain. Thus we can hear without any external ear at all. But in the usual way in which we hear, what we may call accessory apparatus is used, the chief arrangement of which is as follows:—

Passing from the external ear, or pinna, to the back of the nasal cavity is a tubular passsge divided into two parts by a membranous partition, the tympanic membrane. The outer part, or external auditory passage, is an inch and a quarter long. The inner part consists of the tympanic cavity, or middle ear, and the Eustachian tube, which opens into the pharynx, just behind the nasal cavity. In the tympanic cavity are the ossicles, a chain of three little bones

B, Brain. A, Auditory nerve, giving branches to the auditory cells with hair-like processes. E, Endolymph. P, Perilymph. C, Bony capsule of ear. O, Ossicle. T. Tympanic membrane. I, Internal ear.

stretching across the cavity from the tympanic membrane to a hole in the bony capsule surrounding the internal ear. These bones are very delicately adjusted so as to communicate vibrations.

*Mechanism of the Accessory Apparatus.*—The tympanic membrane receives the vibrations of sound, and communicates them to the ossicles; these pass them on to the perilymph, and so to the internal ear. The external ear is of no real use, and we can hear perfectly well without it.

The mode in which sound vibrations are conveyed to the brain is best shown by considering a simpler auditory apparatus than that of man, in whom the organ of hearing is so complicated. For this purpose I propose to take the frog, and in order to make the matter still more simple, to consider it in a comparatively early stage of development, *i.e.*, when all the parts are present, but before they have become complicated. In the frog the essentials are the same, but in place of three ossicles, or small bones connecting the tympanic membrane with the internal ear, there is only one. The above diagram will show clearly how the vibrations of sound are conveyed from the tympanic membrane by the small bone, or ossicle, to a small membrane (called the fenestra ovalis) situated in the bony capsule surrounding the internal ear. By the vibrations of this small membrane the sound is conveyed to the perilymph, internal ear, and endolymph, and so to the auditory cells which are connected with the auditory nerve, and so to the brain. In man the essential principles are the same, but we have three small bones instead of one, and the internal ear is far more complicated. In the frog also the tympanic membrane is on a level with the skin, instead of being situated about an inch away from it down the auditory canal, as in man.

*The Semi-Circular Canals.*—The definite arrangement of these in three planes at right angles to each other is very striking, and was at first supposed to be concerned with the sense of direction of sound. This is probably incorrect. Injury or disease of the canals causes giddiness, headache, and occasionally peculiar rotary movements of the body, but hearing is not affected. It is therefore supposed that they have something to do with the co-ordination of movement and the balancing of the body.

*The Cochlea.*—This is a spiral tube lying in a bony canal, across which it stretches. Its minute structure is too complicated for us to consider in this series of lectures, and the reader must refer to the larger text-books of anatomy. It will suffice to say that running along the spiral cochlea is a structure known as the organ of Corti, which consists of peculiarly modified cells arranged in longitudinal rows, and connected with filaments of the auditory nerve. It is the organ of Corti which is supposed to be concerned in the communication of sounds to the auditory nerve, but in what manner is not exactly determined.

### Appointments.

[It is requested that successful candidates will send a copy of their applications and testimonials, with date of election, to The Editor, The Lodge, Porchester Square, W.]

Plymouth Royal Eye Infirmary.—Miss Edith Olphert has been appointed matron of this hospital. She was trained at the South Devon and East Cornwall Hospital, Plymouth, and was day sister for four and a half years and night sister for six months at the same hospital. Many good wishes accompany Miss Olphert to her new work from her fellow-workers, by whom her departure is heartily regretted.

### Where to Go.

Royal Haymarket Theatre.—Matinée of "Trilby, Monday, December 9th, in aid of the funds of the Royal Eye Hospital, Southwark.

The annual conversazione of the Royal British Nurses' Association will be held on December 9th in the Galleries of the Institute of Painters in Oil Colours, Piccadilly. Tickets and all particulars can be obtained from the Secretary, 17, Old Cavendish Street.

Trained Nurses' Club, 12, Buckingham Street, Strand.—"Massage Talks." On Saturday, November 23rd, nerve cases at a quarter-past two p.m. Admission 6d.

Highbury Athenæum.—Cinderella dances in aid of the funds of the Great Northern Central Hospital on Friday, December 20th; Tuesday, January 21st, 1896; and Thursday, February 27th. Tickets can be obtained of the secretary at the hospital.

Cheyne Hospital, Chelsea.—Exhibition of needlework done by the incurable children, December 3rd and 4th, from two to five p.m.

Trained Nurses' Club.—Annual sale of work, at 12, Buckingham Street, Strand, on December 5th and 6th, from half-past two to half-past nine. Musical and other entertainments.

Brompton Hospital for Consumption.—The usual weekly entertainment on Tuesday evening last was provided by Madame Belle Cole. Madame Belle Cole, who was warmly received, sang in her best manner. Miss Gertrude Izard, Mr. Mandeno Jackson, Mr. Cecil Stephenson, Madamoiselle Henrietta Murkens, Mr. Sydney Brooks, and Miss Lita Jarrett ably assisted her.

### The Half-Trained Nurse.

By a Medical Superintendent.

Can nothing be done to render less ubiquitous half-trained travesties of the genuine nurse? They are the women who wear their outdoor uniform in a manner suggestive of a comic opera; whe put on "side"—so abhorrent to the real nurse—yet, in some cases at least, can barely sign their own names, far less write a legible report; who lack the low voice and quiet manner which are the outward mark of discipline; who insist on discussing the state of the weather while the doctor is examining a case, and are addicted to giving more or less direct hints as to treatment, but cannot be trusted with a clinical thermometer. The picture may seem overdrawn, but I have had exceptional opportunities for studying this undesirable type, and speak, perhaps with feeling, but not without authority. From fever hospitals, private "homes," "special" institutions, workhouses, and asylums these half-trained nurses are pouring into the profession. Has the day not come when a wardmaid, being ambitious, requires only a florid uniform and a knowing bonnet to convert herself into a "nurse"? It was only the other day that an aspirant of this class, duly bedecked, applied at my hospital for a vacancy on the staff. She produced a testimonial from the late medical officer of a fever hospital, stating that, as a wardmaid, she had helped in the nursing work. She left me disconsolate; but I hear she is now private nursing at B——; and is no doubt doing very well, as hard times go!

Have trained nurses nothing to say to this state of things? The evil is widespread—only those having business in the byways of nursing work know how rapidly it is growing. Nor is the tide to be stemmed by raising the status of the trained nurse, although that is a laudable thing in itself. The more highly the trained nurse is esteemed, the more readily will her uniform and calling be adopted by unqualified persons who can find or invent an excuse for doing so. The only feasible method is to deal with the evil at its diverse sources.

Of these sources, the fever hospital is one of the most important—and it is with that I am now concerned. I wish to do the fever nurse no injustice. She is hard-working—often overworked. If she is half-trained it is her misfortune, not her fault. She is often a good nurse in spite of circumstances, but the following facts are true of her in the aggregate: (1) As a probationer she is drawn from a class somewhat lower, as regards education and social status, to that supplying general hospitals; (2) she seldom receives three years' training; (3) the "training" in question not uncommonly amounts to the knowledge she picks up in the wards, although in some large institutions a systematic curriculum, with lectures, has been adopted; (4) she is almost never certificated. That is her position at the best; at the worst, she is a mere "help" engaged during an epidemic, and let loose on the public when it is over. So much for the larger hospitals. The medium-sized institutions, such as are provided by county boroughs, do not possess the organisation necessary to the larger ones, and consequently are not so effective as regards disciplinary training, while the technical knowledge which the probationer-nurse may glean in the wards is, of course, less in proportion to the number of cases. Finally, the wooden or corrugated-iron hospitals of a dozen beds, more or less, provided by rural sanitary authorities, require nurses—and get them. Here the "man and wife" system is in vogue, and cannot but tend to reduce the status of the nursing profession as a whole. To those interested in the question from the nurses' point of view, the report of Dr. Thorne Thorne to the Local Government Board (1880-81, re-issued 1894) on Isolation Hospitals is interesting, if not very pleasing, reading. It is to be feared that things have not much altered since the report was made. I recall one instance in which the laundress of a general hospital was stated to be the nurse for the isolation block! Is it too much to surmise that she has long since discarded the wash-tub for the thermometer?

At present there is a distinct—even an urgent—demand for fever nurses; but, in the nature of the case, it must soon slacken. It is due to the fact that isolation hospitals are springing up all over the country; but such hospitals, in their turn, are "training" nurses. A time will come when no more hospitals will be required. Even now, with a constant demand for her services in fever hospitals, the fever nurse is turning to general work; what will happen when the demand becomes stationary, while she continues to multiply?

Is there a practical remedy? Any plan of reform, to be successful, must recognise the fever nurse as a necessity. General nurses will not, in any considerable number, enter even the larger fever hospitals. In these days of sanitary reform, when hygiene has become a special branch of medical work, requiring a special couse of study, and even a special degree or qualification, is it too much to suggest the constitution of a highly-trained special class of sanitary nurse? In a provincial hospital of the larger class the experiment has been tried. An advertisement for probationers was inserted in The Hospital, and, among the usual shoal of wardmaids and domestic servants, with a sprinkling of better-class applicants, were a sufficient number of the kind required, who were interested in fever work, and attracted by the prospect of systematic training, lectures, and a certificate. They were frankly told of the present state of things, but had common-sense enough to see that their chance of advancement would be all the greater. They now form a staff of which any general hospital might be proud. As regards technical knowledge, apart from anatomy and physiology, their position is exceptional, since they have been taught on the principle that, in addition to the nursing and management of fever cases, they cannot know too much as regards clinical history, pathology, and even medical treatment, so long as they wear their knowledge modestly, and use it only as a guide in following intelligently the instructions of the physician. Here, then, is proof that raw material is obtainable for the production of the sanitary nurse. A scheme of organisation and education on a large scale will be detailed in a subsequent article.

### Notes and Queries.

The contents of the Editor's Letter-box have now reached such unwieldy proportions that it has become necessary to establish a hard and fast rule regarding Answers to Correspondents. In future, all questions requiring replies will continue to be answered in this column without any fee. If an answer is required by letter, a fee of half-a-crown must be enclosed with the note containing the enquiry. We are always pleased to help our numerous correspondents to the fullest extent, and we can trust them to sympathise in the overwhelming amount of writing which makes the new rules a necessity. Every communication must be accompanied by the writer's name and address, otherwise it will receive no attention.

### Queries.

(36) *Lint.*—On which side of lint should ointment be spread? —*J. B.*

(37) *Army Nursing.*—I should be very much obliged by your telling me what steps I should take to obtain an appointment in the Indian Nursing service?—*Nurse.*

(38) *Wedding Gift* —Please tell me whether nurses who do not belong to the Royal National Pension Fund may subscribe to Princess Maud's wedding present?—*S. K.*

(39) *Work.*—Having had three years' training, can you recommend to me a hospital where I might be accepted as an extra nurse? I do not want to enter as a probationer.—*Violet.*

### Answers.

(36) *Lint* (*J. B.*).—On the smoothest side.

(37) *Army Nursing* (*Nurse*).—If you have had three years' training you should get a form of application and a copy of rules from the India Office, St. James's Park.

(38) *Wedding Gift* (*S. K.*)—The gift is to be entirely from policy holders of the Royal National Pension Fund, of which the Princess of Wales is President.

(39) *Work* (*Violet*).—With a certificate for three years' training from a good institution you could take a post as sister or charge nurse in a provincial hospital or in a fever hospital. There is also a demand for trained staff nurses in workhouse infirmaries.

### The Princess Maud Marriage Present Fund.

Nurses sending contributions to this Fund are requested to write outside the envelope in the left-hand corner the words "Princess Maud," as this will save considerable trouble in dealing with the correspondence. Nurses are reminded that all letters on this subject should be addressed to the Manager of The Hospital, 428, Strand, London, W.C., and not to the office of the Pension Fund.

Second List.

Amount previously acknowledged... ... £3 16 0

| | s. | d. |
|---|---|---|
| Nurse Simonsen... | 5 | 0 |
| M. L. Downes ... | 2 | 6 |
| F. Stanally... | 2 | 6 |
| E. Penny ... | 2 | 6 |
| Nurse Vaughan... | 2 | 6 |
| E. H. Dann | 2 | 0 |
| M. I. Tonks | 2 | 0 |
| B. C. Drake | 2 | 0 |
| A. Morton ... | 2 | 0 |
| M. L. Lockhart... | 2 | 0 |
| C. Simmons | 1 | 6 |
| M. A. Ball ... | 1 | 0 |
| M. A. Gibb | 1 | 0 |
| A. M. Stuart | 1 | 0 |
| C. Schlieman | 1 | 0 |
| A. E. Coker | 1 | 0 |
| H. Dickinson | 1 | 0 |
| A. Thorpe ... | 1 | 0 |
| B. Hunter ... | 1 | 0 |
| C. E. Stevens | 1 | 0 |
| S. A. Baker | 1 | 0 |
| E. Lewis | 1 | 0 |
| F. Smith | 1 | 0 |
| F. J. Cozens | 1 | 0 |
| E. Leah | 1 | 0 |
| C. M. Funnell ... | 1 | 0 |
| S. Graham ... | 1 | 0 |
| F. L. Saunders ... | 1 | 0 |
| A. Huzzey ... | 1 | 0 |
| M. Goodwin | 1 | 0 |
| M. Higgins... | 1 | 0 |
| A. Fuller ... | 1 | 0 |
| A. Moore ... | 1 | 0 |
| E. J. Humphreys | 1 | 0 |
| J. Barlow ... | 1 | 0 |
| E. N. ... | 1 | 0 |
| E. Jeffrey ... | 1 | 0 |
| S. Shewery... | 1 | 0 |
| E. M. Grills | 1 | 0 |
| T. C. Steer... | 1 | 0 |
| M. I. ... | 1 | 0 |
| C. S. Phillips ... | 1 | 0 |
| Nurse Kilburn ... | 1 | 0 |
| Nurse Welch ... | 1 | 0 |
| Nurse Evelyn ... | 1 | 0 |
| J. Mercier ... | 1 | 0 |
| A. J. Moorhead... | 1 | 0 |
| E. Ayrton ... | 1 | 0 |
| E. Whitsey... | 1 | 0 |
| J. Gireard ... | 1 | 0 |
| S. J. Teague ... | 1 | 0 |
| A. Cable ... | 1 | 0 |
| A. M. Millington | 1 | 0 |
| K. E. Cleaver ... | 2 | 0 |
| S. L. Cooke ... | 1 | 6 |
| S. C. Borgaes ... | 1 | 6 |
| A. E. Nicholson | 1 | 0 |
| M. L. Damon ... | 1 | 0 |
| S. J. Bellamy ... | 1 | 0 |
| S. Mason ... | 1 | 0 |
| M. A. R. ... | 1 | 0 |
| F. I. Claringbold | 1 | 0 |
| L. Davis ... | 1 | 0 |
| Policy No. 4,192 | 1 | 0 |
| Nurse Freeman... | 1 | 0 |
| Nurse Cotton ... | 1 | 0 |
| S. J. Liles ... | 1 | 0 |
| M. Fraser ... | 1 | 0 |
| S. A. Hobbs ... | 1 | 0 |

| | s. | d. |
|---|---|---|
| E. Cadwallader... | 1 | 0 |
| M. Peck ... | 1 | 0 |
| M. J. Pugh... | 1 | 0 |
| S. S. Stodger ... | 1 | 0 |
| J. L. Palmer ... | 1 | 0 |
| M. Prince ... | 1 | 0 |
| A. Mitchie ... | 1 | 0 |
| S. Carvosso | 1 | 0 |
| A. Turner ... | 1 | 0 |
| E. S. Chapman ... | 1 | 0 |
| R. Trow ... | 1 | 0 |
| E. A. M. ... | 1 | 0 |
| H. Cownley | 1 | 0 |
| F. Shaw ... | 1 | 0 |
| E. Toms ... | 1 | 0 |
| E. E. Williams ... | 1 | 0 |
| J. W. Ronald ... | 1 | 0 |
| N. E. Ratcliffe ... | 1 | 0 |
| S. Bagshaw... | 1 | 0 |
| R. E. Goodwin ... | 1 | 0 |
| L. E. Turner ... | 1 | 0 |
| E. R. Turner ... | 1 | 0 |
| A. G. Mark ... | 1 | 0 |
| A. Garriock ... | 1 | 0 |
| I. C. Sherlock ... | 1 | 0 |
| A. Wakeling ... | 1 | 0 |
| C. A. Millar ... | 1 | 0 |
| M. A. Walters ... | 1 | 0 |
| E. M. Wright ... | 1 | 0 |
| B. Talbot ... | 1 | 0 |
| A. W. ... | 1 | 0 |
| M. A. Soper ... | 1 | 0 |
| A. S. Rimmer ... | 1 | 0 |
| A. Whiteside ... | 1 | 0 |
| E. C. Sluiged ... | 1 | 0 |
| E. Casey ... | 1 | 0 |
| E. Hodgson ... | 1 | 0 |
| M. L. ... | 1 | 0 |
| A. Bridle ... | 1 | 0 |
| J. Kinsley ... | 1 | 0 |
| C. Doran ... | 1 | 0 |
| A Queen's Nurse ... | 1 | 0 |
| Policy No. 1,332, R.N.P.F. ... | 1 | 0 |
| E. E. Kinsley ... | 1 | 0 |
| M. E. Arnold ... | 1 | 0 |
| S. Arnold ... | 1 | 0 |
| H. Arnold ... | 1 | 0 |
| A. Simonds... | 1 | 0 |
| A. Underwood ... | 1 | 0 |
| J. N. Dixon ... | 1 | 0 |
| C. Gower ... | 1 | 0 |
| S. C. R. ... | 1 | 0 |
| J. M. S. ... | 1 | 0 |
| Nurse Hughes ... | 1 | 0 |
| K. Synyer ... | 1 | 0 |
| Nurse Beswick ... | 1 | 0 |
| H. Elsby ... | 1 | 0 |
| Nurse Kent ... | 1 | 0 |
| S. Lambert... | 1 | 0 |
| E. Elgar ... | 1 | 0 |
| J. Huddleston ... | 1 | 0 |
| E. M. Leake ... | 1 | 0 |
| A. Megarry ... | 1 | 0 |
| Nurse Fleetwood | 1 | 0 |
| A. E. P. ... | 1 | 0 |
| M. Barker ... | 1 | 0 |
| A. M. Belton ... | 1 | 0 |
| F. W. Lawrie ... | 1 | 0 |

| | s. | d. |
|---|---|---|
| Nursing Staff, Granville Road Home, Newcastle-on-Tyne | 10 | 0 |
| A. Anderson ... | 2 | 6 |
| E. Gould ... | 2 | 6 |
| A. Goodwin ... | 2 | 6 |
| M. Turner ... | 2 | 0 |
| R. G. Jackson ... | 2 | 0 |
| L. J. Casnati ... | 2 | 0 |
| A. B. ... | 2 | 0 |
| F. Wooll ... | 1 | 0 |
| M. Merrett... | 1 | 0 |
| S. A. Swift ... | 1 | 0 |
| G. Smith ... | 1 | 0 |
| H. J. Moore ... | 1 | 0 |
| E. Stevens ... | 1 | 0 |
| M. Connell... | 1 | 0 |
| P. B. Bloomer ... | 1 | 0 |
| C. E. Spauliotz ... | 1 | 0 |
| M. A. Pettit ... | 1 | 0 |
| C. J. Woodward ... | 1 | 0 |
| M. Church ... | 1 | 0 |
| M. G. Thring ... | 1 | 0 |
| E. H. Gibson ... | 1 | 0 |
| J. Gerrard ... | 1 | 0 |
| K. Sayes ... | 1 | 0 |
| M. M. Lloyd ... | 1 | 0 |
| J. Adam ... | 1 | 0 |
| J. Sutherland ... | 1 | 0 |
| A. Fairlie ... | 1 | 0 |
| Nurse Auld ... | 1 | 0 |
| E. Dark ... | 1 | 0 |
| M. Taylor ... | 1 | 0 |
| E. A. Oxtoly ... | 1 | 0 |
| A. Robinson ... | 1 | 0 |
| S. Adams ... | 1 | 0 |
| T. Mattieson ... | 1 | 0 |
| H. Raven ... | 1 | 0 |
| S. Fox ... | 1 | 0 |
| Nurse Cartwright ... | 1 | 0 |
| E. Chapman ... | 1 | 0 |
| A. M. Watts ... | 1 | 0 |
| B. M. Stowell ... | 1 | 0 |
| H. H. Settle ... | 1 | 0 |
| H. E. E. ... | 1 | 0 |
| E. Stone ... | 1 | 0 |
| L. Wills ... | 1 | 0 |
| H. L. W. ... | 1 | 0 |
| L. King ... | 1 | 0 |
| K. F. A. Goodin ... | 1 | 0 |
| M. L. ... | 1 | 0 |
| E. Jarman ... | 1 | 0 |
| E. Flint ... | 1 | 0 |
| A. L. Bird ... | 1 | 0 |
| A. Bryan ... | 1 | 0 |

| | s. | d. |
|---|---|---|
| M. Kershaw ... | 1 | 0 |
| S. A. Payne ... | 1 | 0 |
| M. McWilliams ... | 1 | 0 |
| J. Brothers ... | 1 | 0 |
| R. Winter ... | 1 | 0 |
| E. Medhurst ... | 1 | 0 |
| A. T. K. ... | 1 | 0 |
| C. E. Goded ... | 1 | 0 |
| M. Bester ... | 1 | 0 |
| S. J. H. F.... | 1 | 0 |
| L. A. Goodacre... | 1 | 0 |
| A. Mackie ... | 1 | 0 |
| Nurse Caunter ... | 1 | 0 |
| A. E. Dryland ... | 1 | 0 |
| H. Pick ... | 1 | 0 |
| J. Lightfoot ... | 1 | 0 |
| E. W. Mowat ... | 1 | 0 |
| M. Millar ... | 1 | 0 |
| M. A. Hill... | 1 | 0 |
| E. Babcock... | 1 | 0 |
| J. Welch ... | 1 | 0 |
| M. S. C. ... | 2 | 6 |
| L. M. G. ... | 5 | 0 |
| J. E. Brown ... | 2 | 6 |
| J. Gibson ... | 2 | 0 |
| E. A. Boyd... | 1 | 0 |
| Miss Charlton ... | 1 | 0 |
| Miss Miller... | 1 | 0 |
| Miss Newson ... | 1 | 0 |
| G. Wood ... | 1 | 0 |
| E. Stanley ... | 1 | 0 |
| E. B. Quarry ... | 1 | 0 |
| M. J. Jenkins ... | 1 | 0 |
| E. M. Smith ... | 1 | 0 |
| E. E. Moore ... | 1 | 0 |
| E. J. Hemm ... | 1 | 0 |
| B. F. Dinwoode ... | 1 | 0 |
| A. F. Smethurst ... | 1 | 0 |
| E. H. ... | 1 | 0 |
| F. J. Daniel ... | 1 | 0 |
| F. E. C. Hodgson ... | 1 | 0 |
| Nurse Hammond ... | 1 | 0 |
| Nurse Brown ... | 1 | 0 |
| A. R. Willis ... | 1 | 0 |
| Miss H. Sargeant ... | 12 | 0 |
| Nurse Barton ... | 1 | 0 |
| Nurse Williams... | 1 | 0 |
| Nurse Norton ... | 1 | 0 |
| Nurse Harding ... | 1 | 0 |
| Nurse Watson ... | 1 | 0 |
| Nurse Beaver ... | 1 | 0 |
| Nurse Goodfellow ... | 1 | 0 |
| Nurse Davies ... | 1 | 0 |
| Policy No 1,393, R.N.P.F. ... | 1 | 0 |

Received per Royal National Pension Fund.

| | s. | d. |
|---|---|---|
| C. Winterbone ... | 2 | 0 |
| E. Todd ... | 2 | 0 |
| J. E. Parsons ... | 1 | 0 |
| J. M. S. Davenport ... | 1 | 0 |
| A. Lawrie ... | 1 | 0 |
| M. C. Palmer ... | 1 | 0 |
| E. K. Collins ... | 1 | 0 |
| A. Page ... | 1 | 0 |
| E. J. Chawner ... | 1 | 0 |
| K. Stilwell ... | 1 | 0 |
| L. E. D. ... | 1 | 0 |
| M. H. S. ... | 1 | 0 |
| E. S. M. Still ... | 1 | 0 |
| Nurse Bristowe... | 1 | 0 |
| H. Preston ... | 1 | 0 |
| C. Tomlinson ... | 1 | 0 |
| E. J. Dennington ... | 1 | 0 |
| A. Cole ... | 1 | 0 |

| | s. | d. |
|---|---|---|
| Nurse Dudden ... | 1 | 0 |
| E. M. Pearce ... | 1 | 0 |
| L. Harris ... | 1 | 0 |
| A. Rolfe ... | 1 | 0 |
| H. E. Hugall ... | 1 | 0 |
| S. Norman ... | 1 | 0 |
| R. Browning ... | 1 | 0 |
| L. A. Smith ... | 1 | 0 |
| M. W. Cross ... | 1 | 0 |
| Nurse Wheeler ... | 1 | 0 |
| S. E. Harrison ... | 1 | 0 |
| E. W. ... | 1 | 0 |
| A. Emen ... | 1 | 0 |
| A. M. C. Burkitt ... | 1 | 0 |
| E. Spiers ... | 1 | 0 |
| A. A. Thompson ... | 1 | 0 |
| Nurse McCulloch ... | 1 | 0 |
| F. Taylor ... | 1 | 0 |

### Presentation.

On leaving the Chelsea Infirmary to take up the duties of matron at Lewisham Infirmary, Miss Lofts was presented by the matron and nurses with a massive silver clasp and handsome tea-table as a mark of their esteem and goodwill.

### The Penrith Fever Hospital.

Whatever may be the beauties and advantages of local government from a political point of view, its working, so far as sanitary matters are concerned, is often disastrous, so little idea do district councillors have of the responsibilities lying upon them. From the account of the performances of the Penrith Joint Hospital Committee as reported in the *Penrith Advertiser*, we gather that the hospital was not originally too well contrived. There are four wards, and two kitchens, together with rooms for the nurse and caretaker (a married couple), and an assistant nurse, and it was reported at the last meeting that there were twenty patients in the hospital suffering from scarlet fever, so we may presume that the total accommodation provided somewhat exceeds this figure. To manage this institution there appear to be a caretaker and a trained nurse (husband and wife), who receive a joint salary of £1 1s. a week. Rations are also supplied, but only during the time that patients are in the hospital. There is also a servant, or, as she is termed, an assistant nurse, who, however, as we are informed, has had no previous experience of fever nursing, and has not been in any hospital before. For the laundry work, cleaning, &c., there are two charwomen at wages of 8s. and 5s. respectively, with rations.

That we may judge of the sort of idea these benighted Penrith Councillors have of the requirements of fever cases let us inquire into the duties of this trained nurse. She has to bake and cook for the whole establishment, and to superintend the rest of the staff in their work, and has to look after her twenty children suffering from scarlet fever. It was stated at the meeting by one of the members "that when cooking had to be done for about thirty people it was not very easy to carry it on with a small range. . . . The nurse had told him that she was baking till eleven o'clock one night." In addition to this, it was stated that the nurses "had had a good deal of night work, which they had done willingly and well." And yet all the time there are members of this petty council who appear to be lost in indignation at "the enormous expenditure" which is going on! The whole matter is a scandal. From our point of view we look with indignation at such misuse of skilled labour, and at the action of men placing a trained nurse in charge of twenty children scattered through various wards, and throwing upon her all the responsibility for their well-being, and then expecting her not only to cook for the whole establishment but to bake as well, and in addition to all this to take her share of looking after these little ones at night.

But from the public point of view the wrong involved in these proceedings is equally great. By the word "hospital" used under the sanction of a public authority, the good people are being deluded into the idea that the patients receive proper hospital treatment, and we have no hesitation in saying that, so far as they are being thereby led to entrust their sick children to the care of the sanitary authorities, they are being deluded by a false pretence. "Hospital treatment" does not mean the occasional attention of a nurse, however well trained, whose thoughts are occupied in housekeeping, whose time is engaged in cooking and baking for "about thirty people," and whose energies are worn out by "a good deal of night work." If any deaths take place under such conditions we should advise an inquest, and in any case we would most strongly urge the heavy responsibility which lies on the Local Government Board in allowing the continuance of such arrangements.

### Wants and Workers.

Spitalfields Area. — Friendly Workers' Interdenominational Committee in connexion with the Mansion House Scheme.—The committee would be glad of the services of one additional lady and one gentleman as friendly workers among the poor in Spitalfields. Already the friendly workers there include a lady nurse and her friend, and we hope that more than one of our readers may be ready to offer themselves for this most interesting work under the chairman's (Mr. Wilkinson) direction. Offers of personal service in this matter should be addressed to the Editor, 428, Strand, W.C.

### Nursing at the Spalding Union Infirmary.

The following account of the discussion on the question of a trained nurse for the Spalding Union appeared in a local paper:—

A Trained Nurse Wanted.

"A rather long discussion took place on the question of appointing a trained nurse, there being a considerable difference of opinion as to whether the person appointed should be simply a trained nurse or qualified as a midwife in addition. Mrs. Pickworth-Farrow, Mr. Wilson, and Mr. Hall held that it was absolutely necessary the nurse should have the qualification named; but the Rev. J. C. Jones, Dr. Davison, the Rev. W. M. Benson, and others opposed the proposal. The Master, on being called into the room, stated that the nurse attended to nearly all such cases. Some of the Guardians replied that the Medical Officer to the Workhouse (Dr. Barritt) was paid for the work, and he ought to perform it.

Dr. Davison said he strongly objected to these half-trained, so-called midwives, whose little knowledge was very dangerous, attending to such cases. He declared that they were not half trained, and that the certificates which they held were bogus.

The Chairman remarked that their late nurse held a midwifery certificate, and he asked whether this was a bogus one.

Dr. Davison replied that he should think so.

After a warm discussion, during which there was a "passage of arms" between the Rev. J. C. Jones and Mr. Hall, a proposition was made by Mr. Lowden, seconded by Mr. Jones, to the effect that the Board advertise for a trained nurse. An amendment was then moved and seconded in favour of a trained nurse who also held a midwifery certificate. On a vote being taken, 10 supported the amendment and 11 voted for the proposition.

The question of salary was next considered. The Rev. W. M. Benson proposed, and Mr. Ouzman seconded, £20 a year, with the usual perquisites.

Mr. Hall moved £25. Dr. Davison seconded.

The Rev. M. H. Marsden then proposed, and Mrs. Pickworth-Farrow seconded, that the amount should be stated as "£20 to £25, with a preference for a nurse holding a midwifery certificate," and this was carried unanimously.

The clerk was instructed to insert the advertisement in two London papers, one being The Hospital."

At a subsequent meeting a letter was read from the Workhouse Nursing Association protesting against the remarks of Dr. Davidson in regard to the midwives' certificate held by Nurse Graham. Later on the Chairman said that if Dr. Davidson had been present he should have asked him to apologise, and that the sweeping accusations made by him against all nurses was very unjustifiable.

### Training at Chelsea Hospital for Women.

The medical staff of the Chelsea Hospital for Women commenced for the first time this summer a course of lectures on elementary anatomy and physiology. At the end of the course a written and oral examination was held by the lecturers, Dr. Arthur Giles and Dr. Eden. The following nurses passed in the first division: Nurses Warland, Cooke, Wilde, and Wilkinson. The rest of the nurses passed in the second class, gaining about two-thirds of the given number of marks.

### Minor Appointments.

Indian Nursing Service.—Miss C. Frances Hill has been appointed Sister on the Indian Nursing Staff, and leaves England next month. She was trained at the Royal Infirmary, Bristol, was sister (of various wards) at Leicester Infirmary for over three years. Miss Hill was then night superintendent for some months at the Metropolitan Hospital, Kingsland Road, and was afterwards sister of St. Barnabas Ward at the Great Ormond Street Children's Hospital for nearly three years. We wish her every success in her new work.

### Everybody's Opinion.

[Correspondence on all subjects is invited, but we cannot in any way be responsible for the opinions expressed by our correspondents. No communications can be entertained if the name and address of the correspondent is not given, or unless one side of the paper only be written on.]

### NURSES' UNIFORMS.

"Nurse Rose" writes: I am indeed pleased to see in your columns a notice with regard to our uniform. I have been very disgusted at the way in which a nurse's uniform is paraded and worn, not only by respectable though not *bonâ fide* persons, but by women whose name one shrinks to mention. I hope, with your correspondent, that something may shortly be done to protect a dress which nurses have hitherto been proud of, and counted as a protection when on district work, &c.

### TRAINING FOR MALE NURSES.

"Ambulance" writes: I am very glad that "One Who Knows" has taken up the subject of training male nurses, and I agree with his advice as to entering the Medical Staff Corps for three years, but there are strict laws regarding enlistment in this corps. I had myself a strong wish to get efficient training as a male nurse, and I passed in everything but eyesight. I had but the slightest dimness in my left eye, which has not caused me any trouble since. I think there is an opening for the training of male nurses if some of the large institutions would just take the matter up. I have the nursing profession at heart, but there seems no opening for training.

### ASYLUM WORKERS.

"W. E. E." writes: May I trespass on your valuable space to say with what great interest I had read recent letters in The Hospital? As a nurse of ten years' experience both in county and private asylums, besides hospitals and an infirmary, I can say that the statements made by the writers are not overdrawn. It is perfectly correct that the doctors are ignorant of the inadequate sleeping accommodation, and "where ignorance is bliss," &c. We work from fourteen to sixteen hours a day, we nearly starve ourselves because we cannot eat the badly cooked food, or else we acquire chronic indigestion by bolting our meals in a few minutes. We suffer the indignities talked of by an "Old Nurse," and if we get half killed are only told that we must "expect that sort of thing" if we work in asylums. If the least mark is found on a patient the unfortunate nurse has to go, characterless, even though, as in many cases, such marks are self-inflicted. The asylum nurse's life is a simple paraphrase on "Stitch, stitch, stitch," for it is work, work, work, from morning to night. None too soon for us has this correspondence been started, for to quote "A Doll," October 19th, "it is only by making known our woes that we may hope to get some redress." With regard to the "Two Asylum Nurses," who wrote also on October 19th, it is very evident, in common with the medical man whose letter was inserted the week before in The Hospital, that they know nothing whatever of what they talk about. In small asylums it rests a great deal with the superintendent how things are conducted, and in a private one where I had the honour (?) to work it was very evident that I, in common with the other nurses, was regarded by the under doctors and housekeeper-matron as a necessary evil; but of the medical superintendent I cannot speak too highly. He was indeed a perfect gentleman—one of Nature's noblemen. Whilst reading the letter of "Nurse K." I could not help exclaiming "hear, hear," at the end of every sentence, and "Nurse K." may rest assured that she has for ever found a warm corner in my heart. I, too, applied for a post, mentioning that I held a certificate, and was told that "nurses" were thought no more of for having it. How things are in "Bonnie Scotland" I know not. God grant they may be better; but as an Irish girl I am proud and happy to say they are far better in "Ould Oireland."

[We shall be glad to hear from "W. E. E." some particular points in which Irish asylum attendants fare better than their English sisters.—Ed. *T. H.*]

### COOKING *VERSUS* NURSING.

"A Matron" writes: I am a trained nurse and experienced matron, and during my whole hospital life I have been struck with the superabundance of applicants for nursing vacancies and the poverty in the supply of cooks. Can nothing be done to persuade foolish girls who under the title of "mother's help" or lady help, do the work of a general servant, to make a bold step, and learn cooking, and thus become valuable and independent members of society? I speak from experience, having been well taught in a good north country home before learning nursing. Lately I had to change my cook in this convalescent home, and have suffered much from the strangers with whom I have tried to fill the vacancy. At last I took my courage in both hands, and dismissed the latest "cook lady," whose mission appeared to be the management of the institution (with an experience perfectly *nil*). Then, with the aid of a young kitchenmaid, I provided good, well-cooked food, with perfect punctuality, and my work "gave great satisfaction." This was all very well for an emergency, but as I worked I thought of the hundreds and thousands of women who wanted work and money, and yet were too proud to avail themselves of such a post, where all heavy work was over by 1.30 p.m., and plenty of time for reading and needlework daily; and three or four times a week opportunity for outdoor exercise. I wish I could convince people that fewer nurses and more cooks are the need of the day.

### "REAL AND SHAM NURSES."

"Brisbane" writes: Apropos of the above, a few weeks ago I saw an advertisement in the *Church Times* for two lady helps, one (as far as I remember) as housemaid, the other as cook. The advertisement stated they were to "wear the uniform of trained nurses." If this sort of thing is to go on nurses will be compelled to wear ordinary dress in self-defence. Some means should be adopted whereby a "real" nurse could be at once distinguished from a sham one. Could not some universal badge be adopted, and patented, and only supplied to those nurses who could bring proof of being properly trained? Soldiers, sailors, and clergymen have a distinctive dress, which is not encroached on by others. Why not nurses? Let "real nurses" be careful lest they bring disrepute on their uniform. Neatness is a great "want" among nurses. I have seen those from recognised institutions sometimes a disgrace to their training school, owing to large fringes, high-heeled shoes, and the untidy way in which their uniform was put on. The nurse who giggles and makes herself conspicuous by her loud voice and laugh when in "'buses" or in the street is not the one I should wish to be at the sick bed of one dear to me. There are many noble exceptions—women whose very look inspires confidence.

### ABUSE OF UNIFORM.

"A Nurse" writes: I have been much interested in the discussion in your valuable paper on nurses' dress. I do not sympathise with many of the nurses, for I think to a certain extent they bring on themselves the slights they complain of. When a nurse takes to curling and frizzing her hair in the fashionable style, and places her bonnet on the top of this erection, it looks anything but a nurse's proper headgear. If she adds to this a not over-clean uniform, finished off with tan shoes, can she wonder at being treated with slight respect? I have worn uniform for many years, and am proud of it, and have always been treated with the greatest respect.

### Novelties for Nurses.

### A NEW FOOD FOR INFANTS.

There are numbers of foods already in the market which are adapted for nursery use, but not so many that the N. D. Food, prepared by John Clapp, of 90, Bishopsgate Within, should not be a welcome addition. The best of babies grow tired of one description of food, and it is therefore an advantage to be able to vary the diet without detriment to the infant. A little addition of sugar will render the food more palatable in some cases. Mr. Clapp's whole meal biscuits are very pleasant and nourishing; they will be welcome in the nursery and throughout the household.

### CONDENSED COFFEE.

The Terrabona Company have sent us samples of coffee essence which they recommend as a substitute for the less convenient forms of the beverage. No condensed coffee could ever equal the best fresh-ground and roasted berry, but it is quite possible to produce a more convenient and a pleasant substitute, and this the Terrabona Company have done. Two teaspoonsful of the condensed coffee added to boiling water will secure a nice cup of coffee without delay. Many nurses would prefer to vary their consumption of tea by an occasional cup of coffee. The ordinary method, however, is too lengthy, and so amongst nurses tea rules alone. This need not be the case if a bottle of Terrabona coffee is kept at hand.

### THE QUEEN CHARLOTTE COLLAR.

A collar, designed by Nurse C. M. Brown, and named "Queen Charlotte," will probably commend itself to some of our readers. It is a narrow, turned-down linen collar, stitched to a high neck band, the softness of which prevents its causing discomfort, and has a small habit shirt to keep it in place. No doubt this novelty will meet with the approval of nurses who object to the ordinary deep turned-down collar. The "Queen Charlotte" is made by Messrs. Debenham and Freebody.

### DRESS NOVELTIES AT MESSRS. GARROULD'S.

The catalogue of nurses' uniforms recently issued by Messrs. Garrould (150, Edgware Road) is of a most valuable and comprehensive nature. Every requirement is anticipated and catered for; indeed it is seldom we have come across anything more complete. The advantage this list possesses to nurses, whose leisure is proverbially scant, and sometimes irregular, cannot be over-estimated. A brief inspection of the varied illustrations will speedily assist the purchaser to make up her mind, and an order can be made out as satisfactorily and with much less trouble than a journey to the premises would entail. The first thing to attract the eye are the bonnets, which are charming both in style and simplicity. We confess to have quite lost our heart to the one which, from its shape, is appropriately named the "Marie Stuart." The quaint little peak in front, so becoming to most faces, is bound with a velvet quilling and relieved by a ruche of white lisse or net. White strings tie under the chin, and a black gauze veil, not, however, we are glad to see, compulsory, hangs behind. The materials employed are all of the best, and the prices not at all out of the way. In cloaks, the "Angelus" deserves a wide popularity. It fits into the figure at the back and is provided with a double flap in front, the underneath one buttoning all the way down from neck to feet, and the upper flaps falling loose over the arms, to which it affords protection. There is a very large and varied assortment of caps, some of which are exceptionally pretty. The new "Sister Dora" is an excellent design and is no trouble to make up, as the drawing of a tape, which lets out for washing, is all that is necessary when it returns from the laundry. The "Pauline" is another nice cap. The peculiarity is a wide upstanding frill all round which makes a becoming framework to the face. There are others too numerous to mention which all have their special attractions. We are glad to observe that dressmaking is one of the features to which attention is called. This is a very great convenience to those who are wanting uniforms in a hurry. Fit and style are both guaranteed, and any class of material can be selected from the number of goods always kept in stock. The serges and cashmeres are all of good quality, and so are the cambrics, galateas, and linens. There are some beautiful designs among the latter, the colours of which are fast and will survive any amount of washing. Aprons are made in every size and quality, and shoes are provided of a shape and material especially suited to nurses, whose feet are apt to suffer from the constant demand made on them. An excellent contrivance for wearing within the boots or shoes of those suffering from flat-foot, is cordially to be recommended, also the wallets and chatelaines, &c., of which space alone forbids us giving a description.

### For Reading to the Sick.

### MINISTRY TO THE SICK.

**Motto.**

"No physician considers his own good in what he prescribes, but the good of the patient."—*Plato.*

**Verses.**

Who is the Angel of the forty days,
To Faith revealing things from sight removed?
Is it not Luke, Physician Heaven-beloved,
The Everlasting Gospel's word his praise?
He in our firmament has lit new rays;
Oh! by his later star illumined, we
The Christ behold. —*Morgan.*

One word of comfort speak to him
Whose brow is dark with care;
One smile for her whose eyes are dim
By sickness or despair;

One look of kind compassion give,
One motion, or a sigh;
One breath to bid the dying live,
One prayer to God on high.
—*Heavenly Thoughts.*

Is it true, O Christ in Heaven,
That the highest suffer most?
* * * * *
That the mark of rank in Nature
Is capacity for pain?
And the anguish of the singer
Makes the sweetness of the strain? —*Anon.*

**Reading.**

"We that are strong ought to bear the infirmities of the weak." Yes, how can we evade or wonder at the claim, since He Himself took our infirmities, and bore our sicknesses; since, though He was rich, yet for our sakes He became poor. . . . Strength and freedom are indeed great gifts; and when a man has realised that they are his, and has thanked God for them, let him turn them to a really great use. Let him exercise and prove them, by stooping down and taking upon himself the burdens of the weak; putting himself in place of the weak; going back, as it were, to stay with them till he can help them onward."—*Dean Paget.*

"Be not slow to visit the sick, for that shall make thee to be beloved."—*Eccls.* vii. 35.

"Honour a Physician, with the honour due unto him, for the Lord hath created him. Give place to the Physician. . . . Let him not go from thee, for thou hast need of him. There is a time when in their hands there is good success. For they shall also pray unto the Lord that He would prosper that which they give for ease and remedy to prolong life."—*Eccls.* xii. 14.

"Heal the sick, and say unto them, 'The Kingdom of God is come nigh unto you.'"—*Luke* x. 9.

## Figures and Tables

**FIG 12 f1:**